# Crystal structure of the Al_20_Mn_5.37_Ni_1.31_ phase in the Al–Mn–Ni system

**DOI:** 10.1107/S2414314621009810

**Published:** 2021-09-24

**Authors:** Qifa Hu, Bin Wen, Changzeng Fan

**Affiliations:** a State Key Laboratory of Metastable Materials, Science and Technology, Yanshan University, Qinhuangdao 066004, People’s Republic of China; Vienna University of Technology, Austria

**Keywords:** crystal structure, high-temperature sinter­ing, *φ* phase, Al–Mn–Ni system

## Abstract

The inter­metallic phase with composition Al_20_Mn_5.37_Ni_1.31_ (icosa­aluminium penta­manganese nickel) was synthesized by high-temperature sinter­ing of a mixture with initial chemical composition Al_60_Mn_7_Ni_3_. Al_20_Mn_5.37_Ni_1.31_ adopts the Co_2_Al_5_ structure type in space-group type *P*6_3_/*mmc*, replacing the Co atoms with the transition-metal atoms Mn and Ni. Structure analysis revealed that one of the two transition-metal sites is partially occupied by Ni [refined occupancy 0.342 (2)] and the other is co-occupied by Mn and Ni with a ratio of 0.895 (14):0.105 (14). The present refined chemical composition is supported by complementary energy-dispersive X-ray fluorescence (EDX) analysis and is in agreement with the previously determined Al–Mn–Ni phase diagram [Balanetskyy *et al.* (2011 ▸). *J. Alloys Compd*, **509**, 3795–3805].

## Abstract 2

The phase Al_20_Mn_5.37_Ni_1.31_ in the ternary system Al–Mn–Ni system was synthesized by high-temperature sinter­ing and its crystal structure has been refined from single-crystal X-ray data.

## Graphical abstract

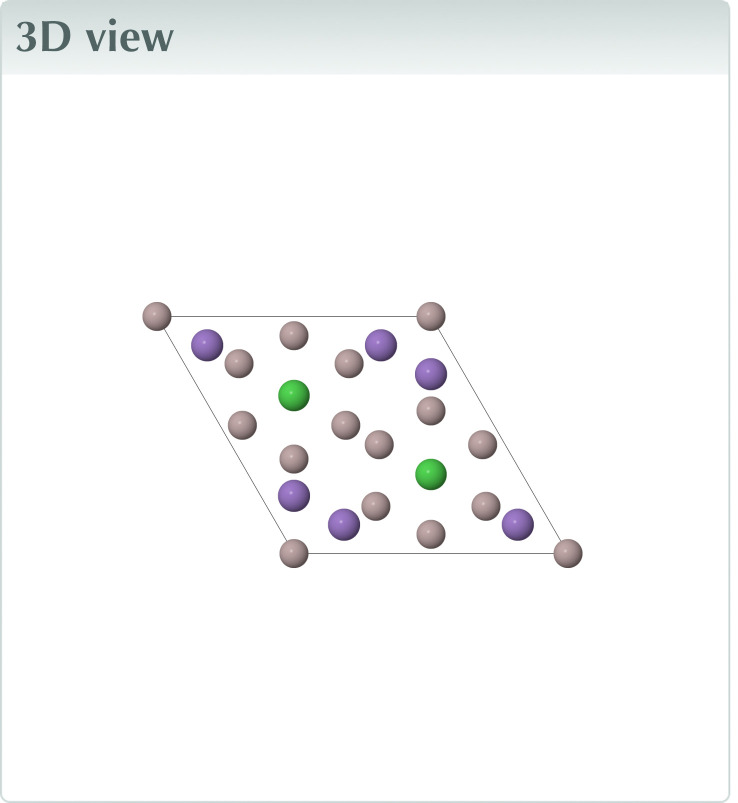

## Structure description

Phases in the ternary Al–Mn–Ni alloy system are structurally complex, also including quasicrystals (QC). For example, an aperiodic diffraction pattern was observed for the alloy with composition Al_60_Mn_11_Ni_4_, exhibiting tenfold rotation symmetry and characterized as a quasi-crystalline phase (Tendeloo *et al.*, 1988 ▸). As a result of their applications in industry, relevant stable and metastable phases in the Al–Mn–Ni system have been investigated thoroughly (Balanetskyy *et al.*, 2011 ▸). Three thermodynamically stable ternary inter­metallics have been reported, among them the *φ* phase adopting the Co_2_Al_5_ structure type [*P*6_3_/*mmc*, *Z* = 4, *a* = 7.6632 (16), *c* = 7.8296 (15) Å; Balanetskyy *et al.*, 2011 ▸]. However, a detailed crystal-structure analysis of the *φ* phase has not been indicated, although its homogeneity chemical composition regions at 1223, 1123, 1023, 973, 918 and 893 K were determined (see Table S1 of the supporting information). It should be noted that such Co_2_Al_5_-type phases have also been found in other systems *e.g.* in the binary Al–Mn system the phase Al_10_Mn_3_ with unit-cell parameters *a* = 7.543, *c* = 7.898 Å (Taylor, 1959 ▸), or the deca­aluminium trinickel iron phase Al_10_Ni_3_Fe_0.83_ that was recently obtained in our group by high-pressure sinter­ing (HPS) of a stoichiometric mixture with nominal composition Al_71_Ni_24_Fe_5_ (Wang *et al.*, 2018 ▸). In the present study, the crystal-structure refinement of a phase with composition Al_20_Mn_5.37_Ni_1.31_ based on single-crystal X-ray diffraction data is reported, in accordance with the SEM/EDX results (see Tables S2 and S3 along with Fig. S1 compiled in the supporting information). This phase is located within the diagram region of the *φ* phase determined previously (see Table S1 of the supporting information).

**Figure 1:**
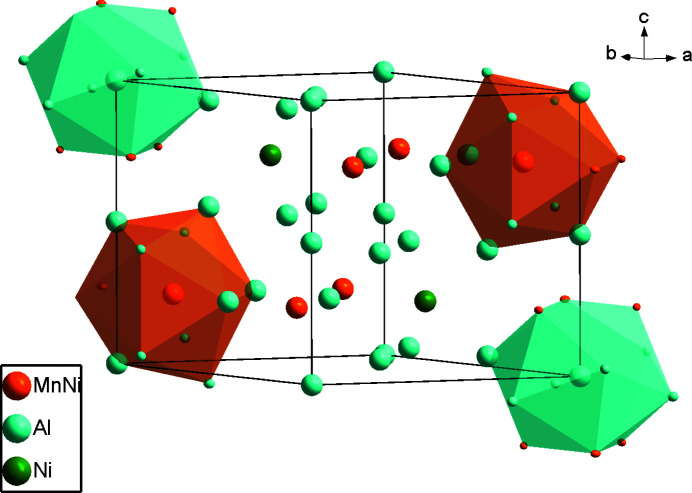
The crystal structure of Al_20_Mn_5.37_Ni_1.31_ with two (Mn1/Ni1) sites and two Al1 atoms displayed with their coordination environments as polyhedra.

**Figure 2:**
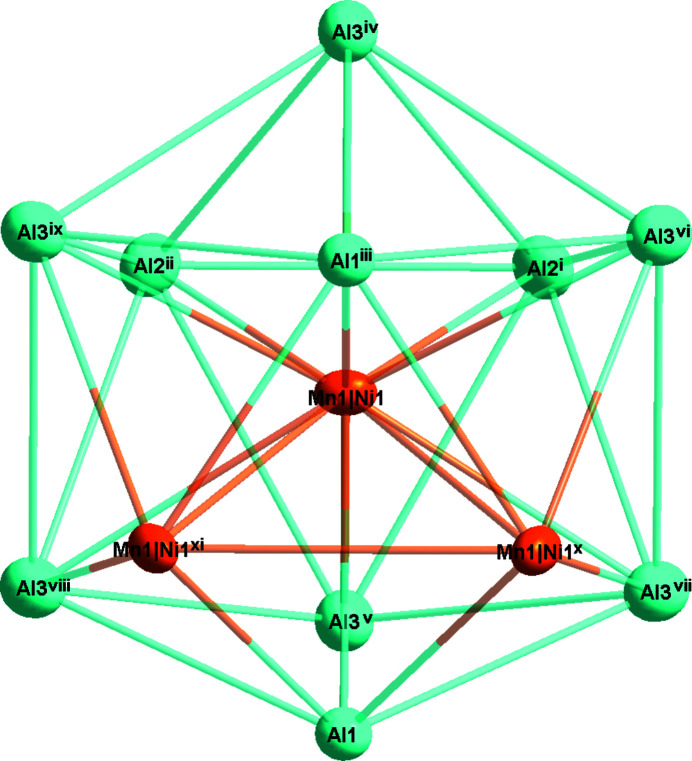
The environment of the (Mn1/Ni1) site. Displacement ellipsoids are given at the 90% probability level. [Symmetry codes: (i) −*x* + *y*, −*x* + 1, *z*; (ii) −*y* + 1, *x* − *y* + 1, *z*; (iii) −*x*, −*y*, *z* + 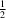 ; (iv) *x*, *y*, −*z* + 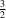 ; (v) *x*, *y*, *z* − 1; (vi) *y*, −*x* + *y*, *z* − 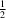 ; (vii) *y*, −*x* + *y*, −*z* + 1; (viii) *x* − *y*, *x*, −*z* + 1; (ix) *x* − *y*, *x*, *z* − 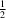 ; (*x*) −*x* + *y*, −*x*, *z*; (xi) −*y*, *x* − *y*, *z*.]

**Figure 3:**
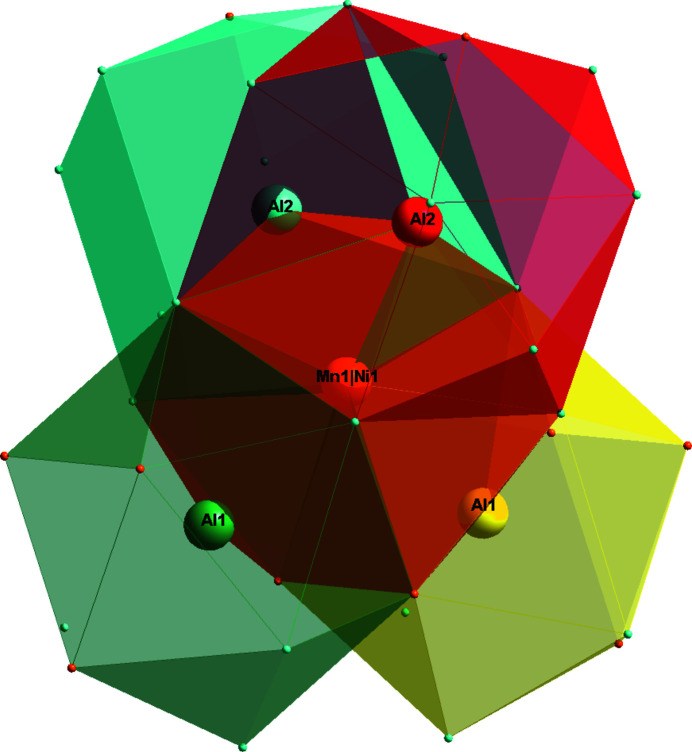
The fusion of five polyhedra centered at one (Mn1/Ni1), two Al1 and two Al2 sites.

With respect to the Co_2_Al_5_ structure type (Newkirk *et al.*, 1961 ▸), in the crystal structure of the Al_20_Mn_5.37_Ni_1.31_ phase the Co atoms are replaced by the transition metals Mn and Ni (Fig. 1 ▸). The asymmetric unit of Al_20_Mn_5.37_Ni_1.31_ comprises five metal sites, three fully occupied by Al atoms at Wyckoff positions 2 *a* (Al1), 6 *h* (Al2) and 12 *k* (Al3), one partially occupied Ni2 site [occupancy 0.342 (2)] at 2 *d* and one co-occupied (Mn1/Ni1) site [occupancy ratio 0.895 (14): 0.105 (14)] at 6 *h*. The environment of the co-occupied (Mn1/Ni1) site is shown in Fig. 2 ▸, where twelve vertices include ten Al atoms (Al1, Al2, Al3) and two symmetry-related (Mn1/Ni1) sites. In the crystal structure, the distorted icosa­hedra centered at Al1 and (Mn1/Ni1) and the polyhedron centered at Al2 are fused with each other, as shown in Fig. 3 ▸.

## Synthesis and crystallization

The high-purity elements Al (indicated purity 99.8%; 2.700 g), Mn (indicated purity 99.96%; 0.6417 g) and Ni (indicated purity 99.9%; 0.2935 g) were mixed in the molar ratio 60:7:3 and ground in an agate mortar. The blended powders were placed into a cemented carbide grinding mound of 9.6 mm diameter and pressed at 4 MPa for about 5 min. The obtained cylindrical block was crushed and a sample with a weight of 50.32 mg was selected and subsequently loaded into a Netzsch STA449C simultaneous thermal analysis apparatus. The sample was heated up to 1373 K for 10 min with a heating rate of 20 K min^−1^. Finally, the sample was slowly cooled to room temperature by turning off the furnace power. Suitable pieces of single-crystal grains were selected from the products for single-crystal X-ray diffraction experiments.

## Refinement

Crystal data, data collection and structure refinement details are summarized in Table 1 ▸. For better comparison with the Co_2_Al_5_ structure type, the labelling scheme and atomic coordinates were adapted from Co_2_Al_5_ (Newkirk *et al.*, 1961 ▸). One of the five metal sites is partially occupied by Ni atoms (Ni2) and one site is co-occupied by Mn and Ni atoms (Mn1/Ni1); all Al atoms show full occupancy. Atoms sharing the same site were constrained to have the same coordinates and anisotropic displacement parameters. The maximum and minimum residual electron densities in the final difference map are located 1.32 Å from the (Mn1/Ni1) site and 0.01 Å from the same site, respectively.

## Supplementary Material

Crystal structure: contains datablock(s) I. DOI: 10.1107/S2414314621009810/wm4153sup1.cif

Structure factors: contains datablock(s) I. DOI: 10.1107/S2414314621009810/wm4153Isup2.hkl

Supporting figures and tables. DOI: 10.1107/S2414314621009810/wm4153sup3.docx

CCDC reference: 2110961

Additional supporting information:  crystallographic information; 3D view; checkCIF report

## Figures and Tables

**Table 1:** Experimental details

Crystal data	
Chemical formula	Al_20_Mn_5.37_Ni_1.31_
*M*_r_	911.74
Crystal system, space group	Hexagonal, *P*6_3_/*m* *m* *c*
Temperature (K)	296
*a*, *c* (Å)	7.6009 (3), 7.8187 (5)
*V* (Å^3^)	391.20 (4)
*Z*	1
Radiation type	Mo *K*α
μ (mm^−1^)	6.85
Crystal size (mm)	0.14 × 0.07 × 0.05

Data collection	
Diffractometer	Bruker D8 Venture Photon 100 CMOS
Absorption correction	Multi-scan (*SADABS*; Krause *et al.*, 2015 ▸)
*T*_min_, *T*_max_	0.588, 0.746
No. of measured, independent and observed [*I* > 2σ(*I*)] reflections	14281, 260, 246
*R*_int_	0.048
(sin θ/λ)_max_ (Å^−1^)	0.715

Refinement	
*R*[*F* ^2^ > 2σ(*F* ^2^)], *wR*(*F* ^2^), *S*	0.016, 0.033, 1.20
No. of reflections	260
No. of parameters	21
Δρ_max_, Δρ_min_ (e Å^−3^)	0.35, −0.46

Computer programs: *APEX3* and *SAINT* (Bruker, 2015 ▸), *SHELXT* (Sheldrick, 2015*a* ▸), *SHELXL* (Sheldrick, 2015*b* ▸), *DIAMOND* (Brandenburg & Putz, 2017 ▸) and *publCIF* (Westrip, 2010 ▸).

## full crystallographic data

### Crystal data

**Table:**

Al_20_Mn_5.37_Ni_1.31_	*D*_x_ = 3.870 Mg m^−^^3^
*M**_r_* = 911.74	Mo *K*α radiation, λ = 0.71073 Å
Hexagonal, *P*6_3_/*m**m**c*	Cell parameters from 5904 reflections
*a* = 7.6009 (3) Å	θ = 3.1–30.5°
*c* = 7.8187 (5) Å	µ = 6.85 mm^−^^1^
*V* = 391.20 (4) Å^3^	*T* = 296 K
*Z* = 1	Fragment, metallic
*F*(000) = 431	0.14 × 0.07 × 0.05 mm

### Data collection

**Table:**

Bruker D8 Venture Photon 100 CMOS diffractometer	246 reflections with *I* > 2σ(*I*)
φ and ω scans	*R*_int_ = 0.048
Absorption correction: multi-scan (*SADABS*; Krause *et al.*, 2015)	θ_max_ = 30.5°, θ_min_ = 3.1°
*T*_min_ = 0.588, *T*_max_ = 0.746	*h* = −10→10
14281 measured reflections	*k* = −10→10
260 independent reflections	*l* = −11→11

### Refinement

**Table:**

Refinement on *F*^2^	21 parameters
Least-squares matrix: full	0 restraints
*R*[*F*^2^ > 2σ(*F*^2^)] = 0.016	*w* = 1/[σ^2^(*F*_o_^2^) + (0.0158*P*)^2^ + 0.1412*P*] where *P* = (*F*_o_^2^ + 2*F*_c_^2^)/3
*wR*(*F*^2^) = 0.033	(Δ/σ)_max_ < 0.001
*S* = 1.20	Δρ_max_ = 0.35 e Å^−^^3^
260 reflections	Δρ_min_ = −0.46 e Å^−^^3^

### Special details

**Table:**

Geometry. All esds (except the esd in the dihedral angle between two l.s. planes) are estimated using the full covariance matrix. The cell esds are taken into account individually in the estimation of esds in distances, angles and torsion angles; correlations between esds in cell parameters are only used when they are defined by crystal symmetry. An approximate (isotropic) treatment of cell esds is used for estimating esds involving l.s. planes.

### Fractional atomic coordinates and isotropic or equivalent isotropic displacement parameters (Å^2^)

**Table:**

	*x*	*y*	*z*	*U*_iso_*/*U*_eq_	Occ. (<1)
Mn1	0.12191 (3)	0.24382 (5)	0.250000	0.00549 (12)	0.895 (14)
Ni1	0.12191 (3)	0.24382 (5)	0.250000	0.00549 (12)	0.105 (14)
Al1	0.000000	0.000000	0.000000	0.0062 (3)	
Al2	0.45923 (6)	0.91845 (12)	0.250000	0.00770 (19)	
Al3	0.19943 (4)	0.39887 (8)	0.93807 (7)	0.00821 (16)	
Ni2	0.666667	0.333333	0.250000	0.0046 (4)	0.342 (2)

### Atomic displacement parameters (Å^2^)

**Table:**

	*U*^11^	*U*^22^	*U*^33^	*U*^12^	*U*^13^	*U*^23^
Mn1	0.00704 (15)	0.00404 (17)	0.00438 (18)	0.00202 (9)	0.000	0.000
Ni1	0.00704 (15)	0.00404 (17)	0.00438 (18)	0.00202 (9)	0.000	0.000
Al1	0.0067 (3)	0.0067 (3)	0.0053 (5)	0.00333 (17)	0.000	0.000
Al2	0.0056 (3)	0.0091 (4)	0.0095 (3)	0.00454 (19)	0.000	0.000
Al3	0.0079 (2)	0.0095 (3)	0.0077 (3)	0.00477 (13)	0.00001 (10)	0.00003 (19)
Ni2	0.0050 (5)	0.0050 (5)	0.0036 (7)	0.0025 (3)	0.000	0.000

### Geometric parameters (Å, º)

**Table:**

Mn1—Al2^i^	2.4251 (3)	Al1—Al3^vi^	2.6698 (5)
Mn1—Al2^ii^	2.4251 (3)	Al2—Ni2^xiv^	2.7310 (8)
Mn1—Al1	2.5292 (2)	Al2—Al3^xv^	2.8132 (6)
Mn1—Al1^iii^	2.5292 (2)	Al2—Al3^xvi^	2.8132 (6)
Mn1—Al3^iv^	2.6438 (6)	Al2—Al3^xvii^	2.8132 (6)
Mn1—Al3^v^	2.6438 (6)	Al2—Al3^xviii^	2.8132 (6)
Mn1—Al3^vi^	2.7236 (5)	Al2—Al2^i^	2.8707 (14)
Mn1—Al3^vii^	2.7236 (5)	Al2—Al2^ii^	2.8707 (14)
Mn1—Al3^viii^	2.7236 (5)	Al2—Al3^xix^	2.9801 (5)
Mn1—Al3^ix^	2.7236 (5)	Al2—Al3^xx^	2.9801 (5)
Mn1—Mn1^x^	2.7799 (6)	Al2—Al3^xxi^	2.9801 (5)
Mn1—Mn1^xi^	2.7799 (6)	Al3—Ni2^xxii^	2.2956 (5)
Al1—Al3^ix^	2.6698 (5)	Al3—Al3^xxiii^	2.7985 (6)
Al1—Al3^xii^	2.6698 (5)	Al3—Al3^xxiv^	2.7985 (6)
Al1—Al3^v^	2.6698 (5)	Al3—Al3^iv^	2.9409 (10)
Al1—Al3^xiii^	2.6698 (5)		
			
Al2^i^—Mn1—Al2^ii^	72.58 (4)	Al3^xv^—Al2—Al2^i^	107.340 (16)
Al2^i^—Mn1—Al1	120.763 (8)	Al3^xvi^—Al2—Al2^i^	107.340 (16)
Al2^ii^—Mn1—Al1	120.763 (8)	Al3^xvii^—Al2—Al2^i^	147.217 (13)
Al2^i^—Mn1—Al1^iii^	120.763 (9)	Al3^xviii^—Al2—Al2^i^	147.217 (13)
Al2^ii^—Mn1—Al1^iii^	120.763 (8)	Mn1^ii^—Al2—Al2^ii^	113.710 (19)
Al1—Mn1—Al1^iii^	101.222 (13)	Mn1^i^—Al2—Al2^ii^	53.710 (19)
Al2^i^—Mn1—Al3^iv^	71.871 (12)	Ni2^xiv^—Al2—Al2^ii^	150.0
Al2^ii^—Mn1—Al3^iv^	71.871 (12)	Al3^xv^—Al2—Al2^ii^	147.217 (13)
Al1—Mn1—Al3^iv^	163.319 (18)	Al3^xvi^—Al2—Al2^ii^	147.217 (13)
Al1^iii^—Mn1—Al3^iv^	62.097 (12)	Al3^xvii^—Al2—Al2^ii^	107.340 (16)
Al2^i^—Mn1—Al3^v^	71.871 (12)	Al3^xviii^—Al2—Al2^ii^	107.340 (16)
Al2^ii^—Mn1—Al3^v^	71.871 (12)	Al2^i^—Al2—Al2^ii^	60.0
Al1—Mn1—Al3^v^	62.097 (12)	Mn1^ii^—Al2—Al3^xix^	57.471 (11)
Al1^iii^—Mn1—Al3^v^	163.319 (18)	Mn1^i^—Al2—Al3^xix^	118.729 (14)
Al3^iv^—Mn1—Al3^v^	134.58 (3)	Ni2^xiv^—Al2—Al3^xix^	105.093 (16)
Al2^i^—Mn1—Al3^vi^	65.942 (17)	Al3^xv^—Al2—Al3^xix^	108.722 (19)
Al2^ii^—Mn1—Al3^vi^	125.493 (18)	Al3^xvi^—Al2—Al3^xix^	57.684 (15)
Al1—Mn1—Al3^vi^	60.965 (11)	Al3^xvii^—Al2—Al3^xix^	151.28 (3)
Al1^iii^—Mn1—Al3^vi^	110.438 (11)	Al3^xviii^—Al2—Al3^xix^	91.228 (9)
Al3^iv^—Mn1—Al3^vi^	122.648 (12)	Al2^i^—Al2—Al3^xix^	61.207 (14)
Al3^v^—Mn1—Al3^vi^	62.829 (9)	Al2^ii^—Al2—Al3^xix^	91.756 (15)
Al2^i^—Mn1—Al3^vii^	65.942 (17)	Mn1^ii^—Al2—Al3^xx^	57.471 (11)
Al2^ii^—Mn1—Al3^vii^	125.493 (18)	Mn1^i^—Al2—Al3^xx^	118.729 (14)
Al1—Mn1—Al3^vii^	110.438 (11)	Ni2^xiv^—Al2—Al3^xx^	105.093 (16)
Al1^iii^—Mn1—Al3^vii^	60.965 (11)	Al3^xv^—Al2—Al3^xx^	57.684 (15)
Al3^iv^—Mn1—Al3^vii^	62.829 (9)	Al3^xvi^—Al2—Al3^xx^	108.722 (19)
Al3^v^—Mn1—Al3^vii^	122.648 (12)	Al3^xvii^—Al2—Al3^xx^	91.228 (9)
Al3^vi^—Mn1—Al3^vii^	65.35 (2)	Al3^xviii^—Al2—Al3^xx^	151.28 (3)
Al2^i^—Mn1—Al3^viii^	125.493 (18)	Al2^i^—Al2—Al3^xx^	61.207 (14)
Al2^ii^—Mn1—Al3^viii^	65.942 (17)	Al2^ii^—Al2—Al3^xx^	91.756 (15)
Al1—Mn1—Al3^viii^	110.438 (11)	Al3^xix^—Al2—Al3^xx^	109.848 (19)
Al1^iii^—Mn1—Al3^viii^	60.965 (11)	Mn1^ii^—Al2—Al3^xxi^	118.729 (14)
Al3^iv^—Mn1—Al3^viii^	62.829 (9)	Mn1^i^—Al2—Al3^xxi^	57.471 (10)
Al3^v^—Mn1—Al3^viii^	122.648 (12)	Ni2^xiv^—Al2—Al3^xxi^	105.094 (16)
Al3^vi^—Mn1—Al3^viii^	167.68 (2)	Al3^xv^—Al2—Al3^xxi^	91.227 (9)
Al3^vii^—Mn1—Al3^viii^	113.20 (2)	Al3^xvi^—Al2—Al3^xxi^	151.28 (3)
Al2^i^—Mn1—Al3^ix^	125.493 (18)	Al3^xvii^—Al2—Al3^xxi^	57.684 (15)
Al2^ii^—Mn1—Al3^ix^	65.942 (17)	Al3^xviii^—Al2—Al3^xxi^	108.722 (19)
Al1—Mn1—Al3^ix^	60.965 (11)	Al2^i^—Al2—Al3^xxi^	91.756 (15)
Al1^iii^—Mn1—Al3^ix^	110.438 (11)	Al2^ii^—Al2—Al3^xxi^	61.207 (14)
Al3^iv^—Mn1—Al3^ix^	122.647 (12)	Al3^xix^—Al2—Al3^xxi^	149.81 (3)
Al3^v^—Mn1—Al3^ix^	62.829 (9)	Al3^xx^—Al2—Al3^xxi^	61.63 (2)
Al3^vi^—Mn1—Al3^ix^	113.20 (2)	Ni2^xxii^—Al3—Mn1^xxviii^	152.54 (3)
Al3^vii^—Mn1—Al3^ix^	167.68 (2)	Ni2^xxii^—Al3—Al1^xxviii^	150.62 (2)
Al3^viii^—Mn1—Al3^ix^	65.35 (2)	Mn1^xxviii^—Al3—Al1^xxviii^	56.843 (13)
Al2^i^—Mn1—Mn1^x^	113.710 (19)	Ni2^xxii^—Al3—Mn1^vi^	99.682 (17)
Al2^ii^—Mn1—Mn1^x^	173.710 (19)	Mn1^xxviii^—Al3—Mn1^vi^	103.866 (18)
Al1—Mn1—Mn1^x^	56.663 (5)	Al1^xxviii^—Al3—Mn1^vi^	55.920 (12)
Al1^iii^—Mn1—Mn1^x^	56.663 (5)	Ni2^xxii^—Al3—Mn1^ix^	99.682 (17)
Al3^iv^—Mn1—Mn1^x^	109.531 (12)	Mn1^xxviii^—Al3—Mn1^ix^	103.866 (18)
Al3^v^—Mn1—Mn1^x^	109.531 (12)	Al1^xxviii^—Al3—Mn1^ix^	55.920 (12)
Al3^vi^—Mn1—Mn1^x^	59.314 (9)	Mn1^vi^—Al3—Mn1^ix^	61.373 (18)
Al3^vii^—Mn1—Mn1^x^	59.314 (9)	Ni2^xxii^—Al3—Al3^xxiii^	125.585 (12)
Al3^viii^—Mn1—Mn1^x^	108.937 (11)	Mn1^xxviii^—Al3—Al3^xxiii^	59.979 (17)
Al3^ix^—Mn1—Mn1^x^	108.937 (11)	Al1^xxviii^—Al3—Al3^xxiii^	58.393 (3)
Al2^i^—Mn1—Mn1^xi^	173.710 (19)	Mn1^vi^—Al3—Al3^xxiii^	57.191 (12)
Al2^ii^—Mn1—Mn1^xi^	113.710 (19)	Mn1^ix^—Al3—Al3^xxiii^	106.707 (14)
Al1—Mn1—Mn1^xi^	56.663 (5)	Ni2^xxii^—Al3—Al3^xxiv^	125.584 (12)
Al1^iii^—Mn1—Mn1^xi^	56.663 (5)	Mn1^xxviii^—Al3—Al3^xxiv^	59.979 (17)
Al3^iv^—Mn1—Mn1^xi^	109.531 (12)	Al1^xxviii^—Al3—Al3^xxiv^	58.393 (3)
Al3^v^—Mn1—Mn1^xi^	109.531 (12)	Mn1^vi^—Al3—Al3^xxiv^	106.707 (14)
Al3^vi^—Mn1—Mn1^xi^	108.937 (11)	Mn1^ix^—Al3—Al3^xxiv^	57.191 (12)
Al3^vii^—Mn1—Mn1^xi^	108.937 (11)	Al3^xxiii^—Al3—Al3^xxiv^	108.69 (2)
Al3^viii^—Mn1—Mn1^xi^	59.314 (9)	Ni2^xxii^—Al3—Al2^xxix^	63.687 (16)
Al3^ix^—Mn1—Mn1^xi^	59.314 (9)	Mn1^xxviii^—Al3—Al2^xxix^	122.461 (14)
Mn1^x^—Mn1—Mn1^xi^	60.0	Al1^xxviii^—Al3—Al2^xxix^	103.515 (17)
Mn1—Al1—Mn1^xxv^	180.0	Mn1^vi^—Al3—Al2^xxix^	51.923 (12)
Mn1—Al1—Mn1^xxvi^	113.326 (11)	Mn1^ix^—Al3—Al2^xxix^	103.968 (17)
Mn1^xxv^—Al1—Mn1^xxvi^	66.674 (10)	Al3^xxiii^—Al3—Al2^xxix^	64.154 (18)
Mn1—Al1—Mn1^xi^	66.674 (10)	Al3^xxiv^—Al3—Al2^xxix^	158.47 (2)
Mn1^xxv^—Al1—Mn1^xi^	113.326 (10)	Ni2^xxii^—Al3—Al2^xxx^	63.685 (16)
Mn1^xxvi^—Al1—Mn1^xi^	180.0	Mn1^xxviii^—Al3—Al2^xxx^	122.460 (14)
Mn1—Al1—Mn1^x^	66.674 (11)	Al1^xxviii^—Al3—Al2^xxx^	103.515 (17)
Mn1^xxv^—Al1—Mn1^x^	113.326 (11)	Mn1^vi^—Al3—Al2^xxx^	103.968 (17)
Mn1^xxvi^—Al1—Mn1^x^	113.326 (10)	Mn1^ix^—Al3—Al2^xxx^	51.923 (12)
Mn1^xi^—Al1—Mn1^x^	66.674 (10)	Al3^xxiii^—Al3—Al2^xxx^	158.47 (2)
Mn1—Al1—Mn1^xxvii^	113.326 (11)	Al3^xxiv^—Al3—Al2^xxx^	64.154 (18)
Mn1^xxv^—Al1—Mn1^xxvii^	66.674 (11)	Al2^xxix^—Al3—Al2^xxx^	114.43 (3)
Mn1^xxvi^—Al1—Mn1^xxvii^	66.674 (10)	Ni2^xxii^—Al3—Al3^iv^	50.167 (13)
Mn1^xi^—Al1—Mn1^xxvii^	113.326 (10)	Mn1^xxviii^—Al3—Al3^iv^	157.292 (14)
Mn1^x^—Al1—Mn1^xxvii^	180.000 (15)	Al1^xxviii^—Al3—Al3^iv^	100.449 (11)
Mn1—Al1—Al3^ix^	63.116 (9)	Mn1^vi^—Al3—Al3^iv^	57.323 (10)
Mn1^xxv^—Al1—Al3^ix^	116.885 (9)	Mn1^ix^—Al3—Al3^iv^	57.323 (10)
Mn1^xxvi^—Al1—Al3^ix^	116.884 (9)	Al3^xxiii^—Al3—Al3^iv^	110.25 (2)
Mn1^xi^—Al1—Al3^ix^	63.116 (9)	Al3^xxiv^—Al3—Al3^iv^	110.25 (2)
Mn1^x^—Al1—Al3^ix^	118.940 (13)	Al2^xxix^—Al3—Al3^iv^	58.486 (11)
Mn1^xxvii^—Al1—Al3^ix^	61.060 (13)	Al2^xxx^—Al3—Al3^iv^	58.486 (11)
Mn1—Al1—Al3^xii^	116.884 (9)	Ni2^xxii^—Al3—Al2^xxxi^	106.471 (18)
Mn1^xxv^—Al1—Al3^xii^	63.115 (9)	Mn1^xxviii^—Al3—Al2^xxxi^	50.658 (11)
Mn1^xxvi^—Al1—Al3^xii^	63.116 (9)	Al1^xxviii^—Al3—Al2^xxxi^	99.196 (16)
Mn1^xi^—Al1—Al3^xii^	116.884 (9)	Mn1^vi^—Al3—Al2^xxxi^	153.83 (2)
Mn1^x^—Al1—Al3^xii^	61.060 (13)	Mn1^ix^—Al3—Al2^xxxi^	113.945 (17)
Mn1^xxvii^—Al1—Al3^xii^	118.940 (13)	Al3^xxiii^—Al3—Al2^xxxi^	104.78 (3)
Al3^ix^—Al1—Al3^xii^	180.0	Al3^xxiv^—Al3—Al2^xxxi^	58.16 (2)
Mn1—Al1—Al3^v^	61.060 (13)	Al2^xxix^—Al3—Al2^xxxi^	142.04 (2)
Mn1^xxv^—Al1—Al3^v^	118.940 (13)	Al2^xxx^—Al3—Al2^xxxi^	88.772 (9)
Mn1^xxvi^—Al1—Al3^v^	63.115 (9)	Al3^iv^—Al3—Al2^xxxi^	144.924 (10)
Mn1^xi^—Al1—Al3^v^	116.885 (9)	Ni2^xxii^—Al3—Al2^xxxii^	106.471 (18)
Mn1^x^—Al1—Al3^v^	116.884 (9)	Mn1^xxviii^—Al3—Al2^xxxii^	50.658 (11)
Mn1^xxvii^—Al1—Al3^v^	63.116 (9)	Al1^xxviii^—Al3—Al2^xxxii^	99.196 (16)
Al3^ix^—Al1—Al3^v^	63.214 (7)	Mn1^vi^—Al3—Al2^xxxii^	113.945 (17)
Al3^xii^—Al1—Al3^v^	116.786 (7)	Mn1^ix^—Al3—Al2^xxxii^	153.83 (2)
Mn1—Al1—Al3^xiii^	118.940 (13)	Al3^xxiii^—Al3—Al2^xxxii^	58.16 (2)
Mn1^xxv^—Al1—Al3^xiii^	61.060 (13)	Al3^xxiv^—Al3—Al2^xxxii^	104.78 (3)
Mn1^xxvi^—Al1—Al3^xiii^	116.885 (9)	Al2^xxix^—Al3—Al2^xxxii^	88.772 (9)
Mn1^xi^—Al1—Al3^xiii^	63.115 (9)	Al2^xxx^—Al3—Al2^xxxii^	142.04 (2)
Mn1^x^—Al1—Al3^xiii^	63.116 (9)	Al3^iv^—Al3—Al2^xxxii^	144.924 (10)
Mn1^xxvii^—Al1—Al3^xiii^	116.884 (9)	Al2^xxxi^—Al3—Al2^xxxii^	57.59 (3)
Al3^ix^—Al1—Al3^xiii^	116.786 (7)	Al3^xxxiii^—Ni2—Al3^xxx^	79.67 (3)
Al3^xii^—Al1—Al3^xiii^	63.214 (7)	Al3^xxxiii^—Ni2—Al3^ix^	134.842 (9)
Al3^v^—Al1—Al3^xiii^	180.000 (14)	Al3^xxx^—Ni2—Al3^ix^	83.37 (2)
Mn1—Al1—Al3^vi^	63.116 (9)	Al3^xxxiii^—Ni2—Al3^xxxiv^	83.37 (2)
Mn1^xxv^—Al1—Al3^vi^	116.884 (9)	Al3^xxx^—Ni2—Al3^xxxiv^	134.842 (9)
Mn1^xxvi^—Al1—Al3^vi^	61.060 (13)	Al3^ix^—Ni2—Al3^xxxiv^	134.842 (9)
Mn1^xi^—Al1—Al3^vi^	118.940 (13)	Al3^xxxiii^—Ni2—Al3^viii^	83.37 (2)
Mn1^x^—Al1—Al3^vi^	63.115 (9)	Al3^xxx^—Ni2—Al3^viii^	134.842 (9)
Mn1^xxvii^—Al1—Al3^vi^	116.885 (9)	Al3^ix^—Ni2—Al3^viii^	79.67 (3)
Al3^ix^—Al1—Al3^vi^	116.786 (7)	Al3^xxxiv^—Ni2—Al3^viii^	83.37 (2)
Al3^xii^—Al1—Al3^vi^	63.214 (7)	Al3^xxxiii^—Ni2—Al3^xxii^	134.842 (9)
Al3^v^—Al1—Al3^vi^	63.214 (7)	Al3^xxx^—Ni2—Al3^xxii^	83.37 (2)
Al3^xiii^—Al1—Al3^vi^	116.786 (7)	Al3^ix^—Ni2—Al3^xxii^	83.37 (2)
Mn1^ii^—Al2—Mn1^i^	167.42 (4)	Al3^xxxiv^—Ni2—Al3^xxii^	79.67 (3)
Mn1^ii^—Al2—Ni2^xiv^	96.289 (19)	Al3^viii^—Ni2—Al3^xxii^	134.842 (9)
Mn1^i^—Al2—Ni2^xiv^	96.290 (19)	Al3^xxxiii^—Ni2—Al2^xxxv^	67.421 (5)
Mn1^ii^—Al2—Al3^xv^	62.135 (13)	Al3^xxx^—Ni2—Al2^xxxv^	67.421 (5)
Mn1^i^—Al2—Al3^xv^	127.69 (2)	Al3^ix^—Ni2—Al2^xxxv^	67.421 (5)
Ni2^xiv^—Al2—Al3^xv^	48.892 (16)	Al3^xxxiv^—Ni2—Al2^xxxv^	140.167 (13)
Mn1^ii^—Al2—Al3^xvi^	62.135 (13)	Al3^viii^—Ni2—Al2^xxxv^	67.421 (5)
Mn1^i^—Al2—Al3^xvi^	127.69 (2)	Al3^xxii^—Ni2—Al2^xxxv^	140.167 (13)
Ni2^xiv^—Al2—Al3^xvi^	48.892 (16)	Al3^xxxiii^—Ni2—Al2^xxxvi^	67.421 (5)
Al3^xv^—Al2—Al3^xvi^	63.03 (2)	Al3^xxx^—Ni2—Al2^xxxvi^	67.421 (5)
Mn1^ii^—Al2—Al3^xvii^	127.69 (2)	Al3^ix^—Ni2—Al2^xxxvi^	140.167 (13)
Mn1^i^—Al2—Al3^xvii^	62.135 (13)	Al3^xxxiv^—Ni2—Al2^xxxvi^	67.421 (5)
Ni2^xiv^—Al2—Al3^xvii^	48.893 (16)	Al3^viii^—Ni2—Al2^xxxvi^	140.167 (14)
Al3^xv^—Al2—Al3^xvii^	65.73 (3)	Al3^xxii^—Ni2—Al2^xxxvi^	67.421 (5)
Al3^xvi^—Al2—Al3^xvii^	97.79 (3)	Al2^xxxv^—Ni2—Al2^xxxvi^	120.0
Mn1^ii^—Al2—Al3^xviii^	127.69 (2)	Al3^xxxiii^—Ni2—Al2^ii^	140.167 (13)
Mn1^i^—Al2—Al3^xviii^	62.135 (13)	Al3^xxx^—Ni2—Al2^ii^	140.167 (13)
Ni2^xiv^—Al2—Al3^xviii^	48.893 (16)	Al3^ix^—Ni2—Al2^ii^	67.421 (5)
Al3^xv^—Al2—Al3^xviii^	97.79 (3)	Al3^xxxiv^—Ni2—Al2^ii^	67.421 (5)
Al3^xvi^—Al2—Al3^xviii^	65.73 (3)	Al3^viii^—Ni2—Al2^ii^	67.421 (5)
Al3^xvii^—Al2—Al3^xviii^	63.03 (2)	Al3^xxii^—Ni2—Al2^ii^	67.421 (5)
Mn1^ii^—Al2—Al2^i^	53.710 (19)	Al2^xxxv^—Ni2—Al2^ii^	120.0
Mn1^i^—Al2—Al2^i^	113.711 (19)	Al2^xxxvi^—Ni2—Al2^ii^	120.0
Ni2^xiv^—Al2—Al2^i^	150.0		

Symmetry codes: (i) −*y*+1, *x*−*y*+1, *z*; (ii) −*x*+*y*, −*x*+1, *z*; (iii) −*x*, −*y*, *z*+1/2; (iv) *x*, *y*, −*z*+3/2; (v) *x*, *y*, *z*−1; (vi) *x*−*y*, *x*, −*z*+1; (vii) *x*−*y*, *x*, *z*−1/2; (viii) *y*, −*x*+*y*, *z*−1/2; (ix) *y*, −*x*+*y*, −*z*+1; (x) −*y*, *x*−*y*, *z*; (xi) −*x*+*y*, −*x*, *z*; (xii) −*y*, *x*−*y*, *z*−1; (xiii) −*x*, −*y*, −*z*+1; (xiv) *x*, *y*+1, *z*; (xv) *y*, −*x*+*y*+1, *z*−1/2; (xvi) *y*, −*x*+*y*+1, −*z*+1; (xvii) *x*−*y*+1, *x*+1, *z*−1/2; (xviii) *x*−*y*+1, *x*+1, −*z*+1; (xix) −*x*+*y*, −*x*+1, *z*−1; (xx) −*x*+*y*, −*x*+1, −*z*+3/2; (xxi) −*y*+1, *x*−*y*+1, −*z*+3/2; (xxii) −*x*+1, −*y*+1, −*z*+1; (xxiii) *x*−*y*, *x*, −*z*+2; (xxiv) *y*, −*x*+*y*, −*z*+2; (xxv) −*x*, −*y*, −*z*; (xxvi) *x*−*y*, *x*, −*z*; (xxvii) *y*, −*x*+*y*, −*z*; (xxviii) *x*, *y*, *z*+1; (xxix) *y*−1, −*x*+*y*, −*z*+1; (xxx) *x*−*y*+1, *x*, −*z*+1; (xxxi) −*x*+*y*, −*x*+1, *z*+1; (xxxii) −*y*+1, *x*−*y*+1, *z*+1; (xxxiii) *x*−*y*+1, *x*, *z*−1/2; (xxxiv) −*x*+1, −*y*+1, *z*−1/2; (xxxv) *x*, *y*−1, *z*; (xxxvi) −*y*+2, *x*−*y*+1, *z*.
