# Supplementary material for: Crystal structure of the Al20Mn5.37Ni1.31 phase in the Al–Mn–Ni system
Source: IUCrdata. 2021 Sep 24;6(Pt 9):x210981. doi: 10.1107/S2414314621009810 (PMC9462369; doi:10.1107/S2414314621009810)
Supplement: Supplementary file 3 [file x-06-x210981-sup3.docx]

**SUPPLEMENTARY MATERIALS:**

**Crystal structure of the Al_20_Mn_5.37_Ni_1.31_ phase in Al-Mn-Ni system**

**Qifa Hu, Bin Wen and Changzeng Fan***

State Key Laboratory of Metastable Materials Science and Technology, Yanshan University,

Qinhuangdao 066004, P.R. China

*Correspondence email: [chzfan@ysu.edu.cn](mailto:chzfan@ysu.edu.cn)

**Tab. S1** The homogeneity region of ϕ at different temperatures from reference [1]

| Temperature（K） | The proportion of Al, Mn and Ni (%) | | | |
| --- | --- | --- | --- | --- |
| 1223 | 71.5 : 23.3 : 5.0 | 74.4 : 22.6 : 3.0 | 72.5 : 15.3 : 12.2 | 69.2 : 21.2 : 9.6 |
| 1123 | 73.0 : 23.4 : 3.6 | 77.5 : 19.8 : 2.7 | 74.1 : 12.5 : 13.4 | 69.4 : 20.0 : 10.6 |
| 1023 | 72.5 : 22.2 : 5.3 | 76.9 : 21.1 : 2.0 | 73.6 : 14.2 : 12.2 | 69.3 : 21.1 : 9.6 |
| 973 | 77.0 : 21.5 : 1.5 | 75.2 : 15.7 : 9.1 | 74.0 : 14.5 : 11.5 |  |
| 918 | 76.8 : 21.4 : 1.8 | 74.9 : 15.6 : 9.5 |  |  |
| 893 | 76.8 : 21.4 : 1.8 | 74.9 : 15.6 : 9.5 |  |  |

Tab. S2 shows different models with the Al, Mn and Ni atoms refined at different positions. The standard deviation (, x_i_ measures the percentage of different elements used in structural refinement, u_i_ shows the average percentage of different elements obtained by the EDS analysis shown in Tab. S3) is used as an indicator of the refinement quality. The reported refinement choice (in bold) is the most suitable one to fit the EDS averaged results (Al : Mn : Ni = 79.47 : 16.14 : 4.39).

| location | | compositions | | | Standard Deviation | R1 |
| --- | --- | --- | --- | --- | --- | --- |
| 2*d* | 6*h* | Al | Mn | Ni |  |  |
| Partial Mn | Mn/Ni | 74.63 | 23.05 | 2.32 | 5.02 |  |
| Partial Ni | Mn | 74.97 | 22.49 | 2.33 | 4.65 |  |
| Partial Ni | Mn/Ni | **74.95** | **20.13** | **4.91** | **3.49** | **1.6** |
| Partial Ni | Mn/Al | Occupancy error | | |  |  |
| Partial Al | Mn/Ni | 78.29 | 19.30 | 2.41 | 2.26 | 1.7 |
| Al/Ni | Mn | Occupancy error | | |  |  |
| Al/Mn | Mn | Occupancy error | | |  |  |

**Tab. S2** Different choices of refinement and the resulting refined chemical compositions

In order to guide the crystal structure refinement process, the chemical compositions were examined quantitatively by energy dispersive X-ray spectroscopy (EDS) analysis attached to a Hitachi S-3400N SEM. The examined points are designated in Fig. S1 and the corresponding results are listed in Tab. S3. The fluctuation of chemical composition for different points is probably caused by the tilt of the single crystal surface to the incident beam. In addition, the conductive adhesives and glues may also result in the detected impurity elements of carbon. For ease of viewing, the proportions of Al, Mn and Ni are calculated and shown in last column of the Tab.S3.


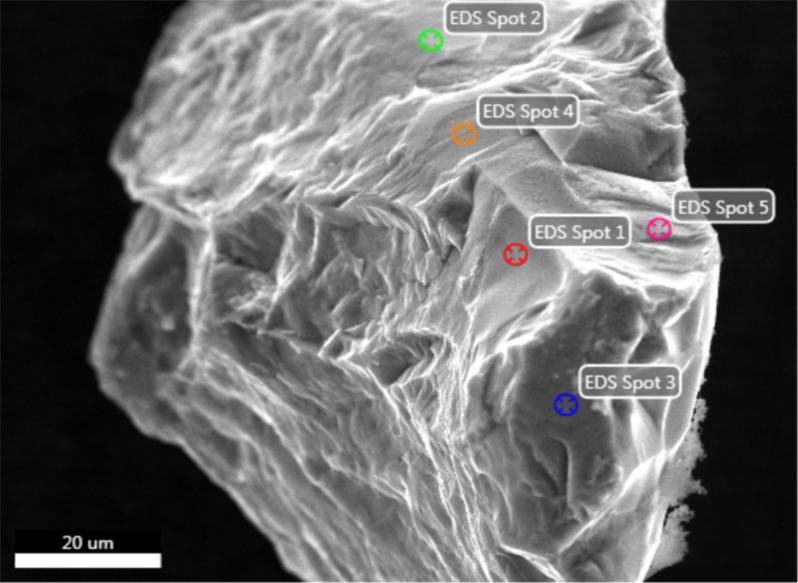


**Fig. S1** single crystal of Al_21.64_Mn_5.38_Ni_0.62_

**Tab. S3** EDS results for selected points as designated in Fig. S1

|  | Element | Weight(%) | Atomic(%) | Error(%) | Al:Mn:Ni |
| --- | --- | --- | --- | --- | --- |
| Spot 1 | C K | 35.43 | 58.20 | 10.26 | 75.3:19.9:4.8 |
|  | O K | 6.70 | 8.26 | 10.95 |  |
|  | AlK | 34.50 | 25.23 | 4.63 |  |
|  | MnK | 18.54 | 6.66 | 2.31 |  |
|  | NiK | 4.83 | 1.62 | 4.63 |  |
| Spot 2 | C K | 42.78 | 63.10 | 9.85 | 82.5:14.0:3.5 |
|  | O K | 9.43 | 10.45 | 10.51 |  |
|  | AlK | 33.23 | 21.82 | 3.99 |  |
|  | MnK | 11.48 | 3.70 | 2.56 |  |
|  | NiK | 3.07 | 0.93 | 5.13 |  |
| Spot 3 | AlK | 63.78 | 78.47 | 5.11 | 78.5:16.4:5.1 |
|  | MnK | 27.17 | 16.42 | 2.39 |  |
|  | NiK | 9.05 | 5.12 | 4.41 |  |
| Spot 4 | C K | 35.82 | 57.59 | 10.21 | 78.2:17.5:4.3 |
|  | O K | 8.20 | 9.89 | 10.42 |  |
|  | Al K | 35.54 | 25.43 | 4.41 |  |
|  | Mn K | 16.20 | 5.69 | 2.33 |  |
|  | NiK | 4.24 | 1.39 | 4.74 |  |
| Spot 5 | C K | 42.21 | 63.06 | 9.79 | 79.2:16.0:4.8 |
|  | O K | 9.56 | 10.70 | 10.44 |  |
|  | Al K | 31.23 | 20.77 | 4.25 |  |
|  | Mn K | 12.86 | 4.20 | 2.43 |  |
|  | NiK | 4.16 | 1.27 | 4.46 |  |

[1] Balanetskyy, S., Meisterernst, G., Grushko, B. & Feuerbacher, M. (2011). *Journal of Alloys & Compounds*. **509** (9),3795-3805.
